# Serologic Markers in Relation to Parasite Exposure History Help to Estimate Transmission Dynamics of *Plasmodium vivax*

**DOI:** 10.1371/journal.pone.0028126

**Published:** 2011-11-29

**Authors:** Fadile Yildiz Zeyrek, Nirianne Palacpac, Fehmi Yuksel, Masanori Yagi, Kaori Honjo, Yukiko Fujita, Nobuko Arisue, Satoru Takeo, Kazuyuki Tanabe, Toshihiro Horii, Takafumi Tsuboi, Ken J. Ishii, Cevayir Coban

**Affiliations:** 1 Laboratory of Malaria Immunology, Immunology Frontier Research Center, Osaka University, Osaka, Japan; 2 Department of Microbiology, Harran University School of Medicine, Sanliurfa, Turkey; 3 Department of Molecular Protozoology, Research Institute for Microbial Diseases, Osaka University, Osaka, Japan; 4 Global Collaboration Center, Osaka University, Osaka, Japan; 5 Cell-Free Science and Technology Research Center, Ehime University, Ehime, Japan; 6 Laboratory of Malariology, International Research Center of Infectious Diseases, Research Institute for Microbial Diseases, Osaka University, Osaka, Japan; 7 Laboratory of Vaccine Science, Immunology Frontier Research Center, Osaka University, Osaka, Japan; Université Pierre et Marie Curie, France

## Abstract

*Plasmodium vivax* infection has been gaining attention because of its re-emergence in several parts of the world. Southeastern Turkey is one of the places in which persistent focal malaria caused exclusively by *P. vivax* parasites occurs. Although control and elimination studies have been underway for many years, no detailed study has been conducted to understand the mechanisms underlying the ineffective control of malaria in this region. Here, for the first time, using serologic markers we try to extract as much information as possible in this region to get a glimpse of *P. vivax* transmission. We conducted a sero-immunological study, evaluating antibody responses of individuals living in Sanliurfa to four different *P. vivax* antigens; three blood-stage antigens (PvMSP1_19_, PvAMA1-ecto, and PvSERA4) and one pre-erythrocytic stage antigen (PvCSP). The results suggest that a prior history of malaria infection and age can be determining factors for the levels and sustainability of naturally acquired antibodies. Significantly higher antibody responses to all the studied antigens were observed in blood smear-negative individuals with a prior history of malaria infection. Moreover, these individuals were significantly older than blood smear-negative individuals with no prior history of infection. These data from an area of sole *P. vivax*-endemic region may have important implications for the global malaria control/elimination programs and vaccine design.

## Introduction

*Plasmodium vivax* parasites are the main cause of widespread human malaria infections, with at least 35–80 million cases reported each year [1], [2]. Although neglected for a long time, the morbidity and public health burden caused by *P. vivax* is very high, resulting in renewed attention [3], [4], [5]. There is a general consensus that acquired immunity to *P. vivax* is age-dependent and develops more quickly than immunity to *P. falciparum* [5], [6], [7], [8]. However, *P. vivax* parasites have distinct biological features, such as persistence in the host due to hypnozoites/relapses, effective gametocyte production, and rapid and efficient adaptation to mosquitoes [9]. Therefore, several questions remain to be addressed before an effective vaccine can be developed: What are the antigen(s) eliciting protective immune responses? What are the immunological correlates for protection (e.g. serum immunoglobulins such as *P. vivax*-specific IgG and IgM, cytokines and chemokines)? What are the host age groups? What are the kinetics of acquired immunity against *P. vivax*? [4]. To date, analysis of *P. vivax* infection has been hampered by the fact that the majority of studies analyzing serological parameters and disease severity have been performed in patients co-infected with *P. falciparum* [6], [7], [10], [11], [12], [13], [14].

Southeastern Turkey is one of the few regions in which *P. vivax* is the only *Plasmodium* species present [15], [16], [17]. Despite extensive control measures, an increasing number of malaria cases have been recorded since the early 1990s. We recently undertook a sero-epidemiological investigation to understand the endemicity and host immune responses to *P. vivax* in Sanliurfa province [16]. In an earlier study, we observed increased sero-reactivity (∼54% IgG positivity) to the C-terminal region of the PvMSP1_19_ antigen (one of the leading candidate vaccine antigens) in serum samples from infected individuals [16], and a further study showed limited and unique *pvmsp1* polymorphisms in the parasite population in Turkey [17]. Therefore, comprehensive studies are needed to completely understand the immune responses to *P. vivax* antigens in this region. Such an understanding will facilitate the design of effective vaccination strategies against *P. vivax* [9].

As a first step, in the present study, we analyzed naturally acquired antibody responses to four *P. vivax* antigens that may be potential vaccine candidates. Total IgM and IgG antibody responses (including IgG subclasses) to three blood-stage antigens, PvMSP1_19_, PvAMA1-ecto and PvSERA4, and one pre-erythrocytic stage antigen, PvCSP, were examined in individuals living in Sanliurfa. Any association between antibody responses to each of the *P. vivax* antigens and the degree of parasitemia in the patients was investigated. Baseline data provides important information for the elimination programs targeting vivax malaria by drugs or vaccines.

## Results

### Naturally acquired antibody responses to pre-erythrocytic and erythrocytic antigens

Although the vivax malaria elimination program was launched in 1925 [18], there is no clear information regarding the endemicity rates and transmission dynamics in southeastern Turkey. According to the WHO, *P. vivax* transmission was reported in seven Turkish provinces in 2006, and 84% of cases occurred in the southeastern cities of Diyarbakir and Sanliurfa [17]. The Sanliurfa region, which accounts for the majority of malaria cases, has recently been studied [16], [17]. For the present study, 195 serum samples were collected from the towns of Siverek and Harran.

The naturally acquired immune responses to the four *P. vivax* candidate vaccine antigens (PvMSP1_19_, PvAMA1-ecto and PvSERA4 and PvCSP) were examined and total IgM and IgG antibody responses (including IgG subclasses) were measured. In the total study population (*n* = 195), 79.1% individuals were seropositive for either IgG or IgM against at least one of the four antigens studied, while 62.1% were seropositive for IgG alone, and 65.6% were seropositive for IgM alone (Table 1). IgG responses to PvMSP1_19_ were seen most frequently in 50.3% of individuals, followed by responses to PvCSP (33.8%), PvAMA1-ecto (21%) and PvSERA4 (16.4%). However, IgM antibody responses to PvSERA4 and PvCSP were observed in the majority of individuals (49.2% and 36.9%, respectively) (Table 1). The IgG subclasses observed in the IgG responders were mainly IgG1, followed by IgG3. The IgG3 responses to PvCSP were more prevalent than those to PvSERA4. In addition, IgG2 and IgG4 responses to PvSERA4 were seen more frequently (in 15.6% and 12.5% of patients, respectively) than to the other antigens (Table 1). Taken together, these results show > 60% IgG seroprevalence to tested *P. vivax* antigens in individuals living in the Sanliurfa region. Interestingly, despite the acknowledged lack of sensitivity in estimating malaria transmission using IgM responses [19], at the present data-set 35–50% of the samples showed IgM responses to at least one of the antigens (particularly to PvCSP and PvSERA4).

**Table 1:** Anti-*P. vivax* antibody prevalence in Sanliurfa.

Antigen	Antibody responses (n = 195)Number (%)			IgG subclasses among IgG responders (%)				
	IgG	IgM	IgG+IgM	IgG1	IgG2	IgG3	IgG4	
**PvMSP1**	98 (50.3%)	55 (28.2%)	105 (53.6%)	93.9	11.2	70.4	5.1	
**PvAMA1-ecto**	41 (21.0%)	35 (17.9%)	66 (33.7%)	95	7.5	77.5	7.5	
**PvSERA4**	32 (16.4%)	96 (49.2%)	106 (54.1%)	90.6	15.6	59.4	12.5	
**PvCSP (chimeric)**	66 (33.8%)	72 (36.9%)	88 (45%)	76.9	6.2	86.2	16.9	
**Total**	121 (62.1%)	128 (65.6%)	155 (79.1%)					

### Antibody responses in relation to a prior history of malaria infection

**Figure 1:**
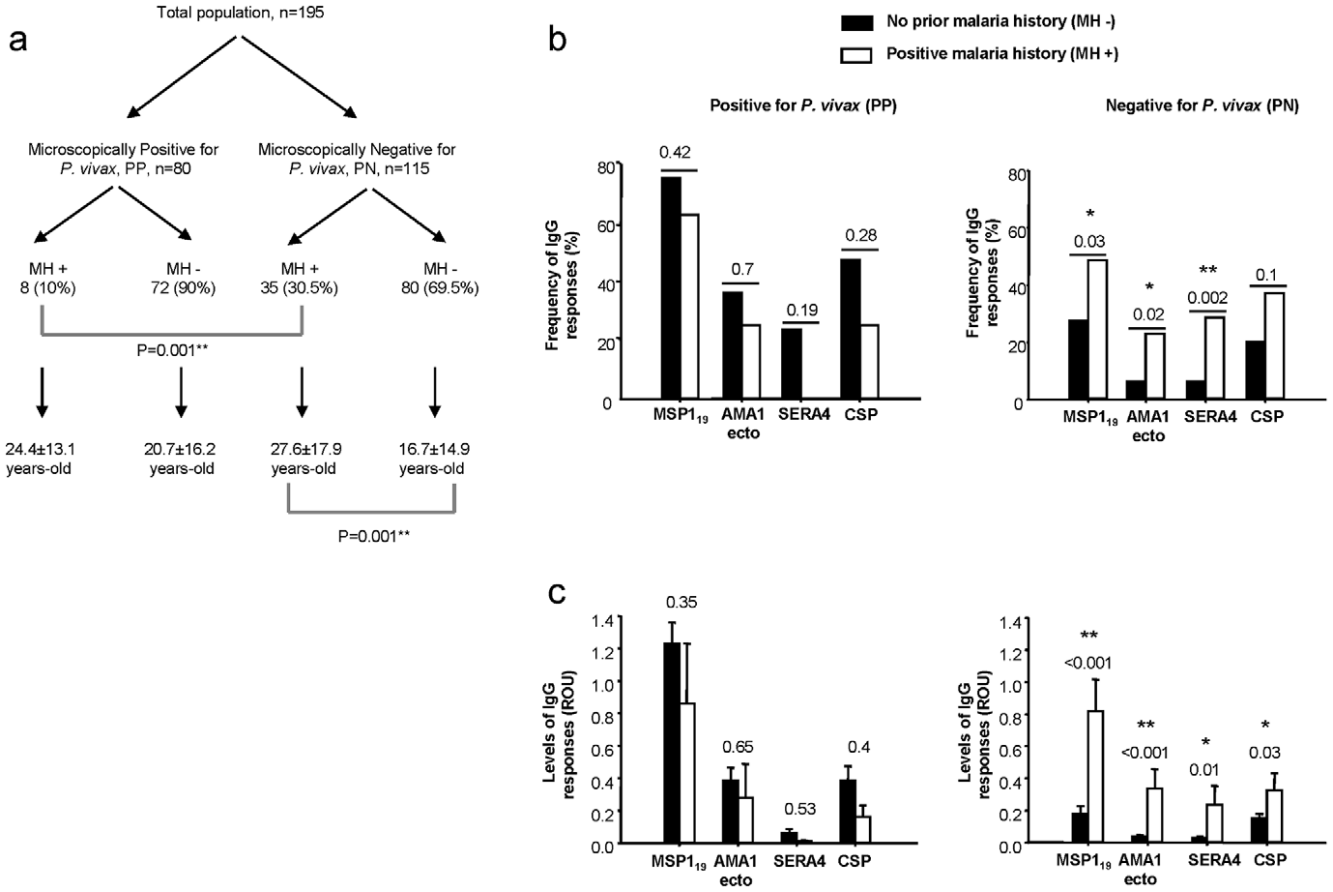
Impact of malaria infection history on naturally acquired antibody responses against *P. vivax* antigens. **(a)** Malaria infection history (MH) and its relationship to age. The total population was categorized according to blood smear positive (PP) and negative (PN) status for *P. vivax*, and then according to malaria infection history and age. The frequency (percentage seropositivity) **(b)** and serum antibody levels (ROU) **(c)** of the total IgG responses against the four antigens are depicted. The χ^2^ test was used to compare the frequencies and mean ages between groups **(a)**. Bars **(c)** represent the mean±SEM, and ROU levels were compared using multiple comparison analysis in conjunction with ANOVA (*P<0.05 and **P<0.01).

Although 2000–6000 cases of malaria were recorded annually in Sanliurfa up until 2003, the incidence declined rapidly after the government's efforts to control the disease using chloroquine and primaquine [17]. According to the Annual Report of the Sanliurfa National Malaria Control Center, the incidence of parasite infection in 2002, 2004 and 2008 was 12.96, 3.20 and 1.85 per 1000 population, respectively [17], and the number of *P. vivax* cases decreased by about 86% from 2002 to 2008 and by 42% from 2004 to 2008. Based on this information that the infection rate had decreased dramatically over the last decade, we examined whether there may be any difference in the antibody responses between younger (< 6 years old) and older individuals. The study population was grouped according to their history of malaria infection (Figure 1a). The results showed that 10% of PP (parasite positive) individuals had a history of malaria infection, but this rose to 30.5% in PN (parasite negative) individuals (χ^2^ = 11.195; *P* = 0.001). Importantly, the mean age of PN individuals with a history of malaria infection was significantly higher than that of individuals with no prior history of infection (27.6±17.9 years *vs*. 16.7±14.9 years; *P* = 0.001, t = -3.383). Thus, these observations suggest that in this region, it appears that repeated exposure to malaria correlates with significantly higher percentage of PN individuals.

We next analyzed the naturally acquired antibody responses to the four candidate antigens according to each individual's infection history. Primary *P. vivax* infections evoked high IgG responses to all four antigens, both in terms of frequency and serum concentration in the PP group with no history of malaria infection (Figure 1b and 1c). In cases of re-infection (which occurred in 10% of the PP group; Figure 1a), neither the prevalence nor the level of the IgG responses to the four antigens changed (Figure 1b and 1c). It is, however, noteworthy that IgG antibody levels to PvSERA4 were either very low or undetectable after re-infection. In contrast, PN individuals with a history of malaria infection showed higher IgG responses to all four antigens both in terms of frequency and serum levels (*P<0.05 and **P<0.01; Figure 1b and 1c). However, there was a substantial IgG response, but a very low response to PvMSP1_19_ and PvCSP in those PN individuals with no prior history of malaria infection. When combined, these results give important clues on the likely relationship of previous malaria exposure and seroconversion to malaria antigens tested.

### Correlation between antibody frequency, serum concentration and age

**Figure 2:**
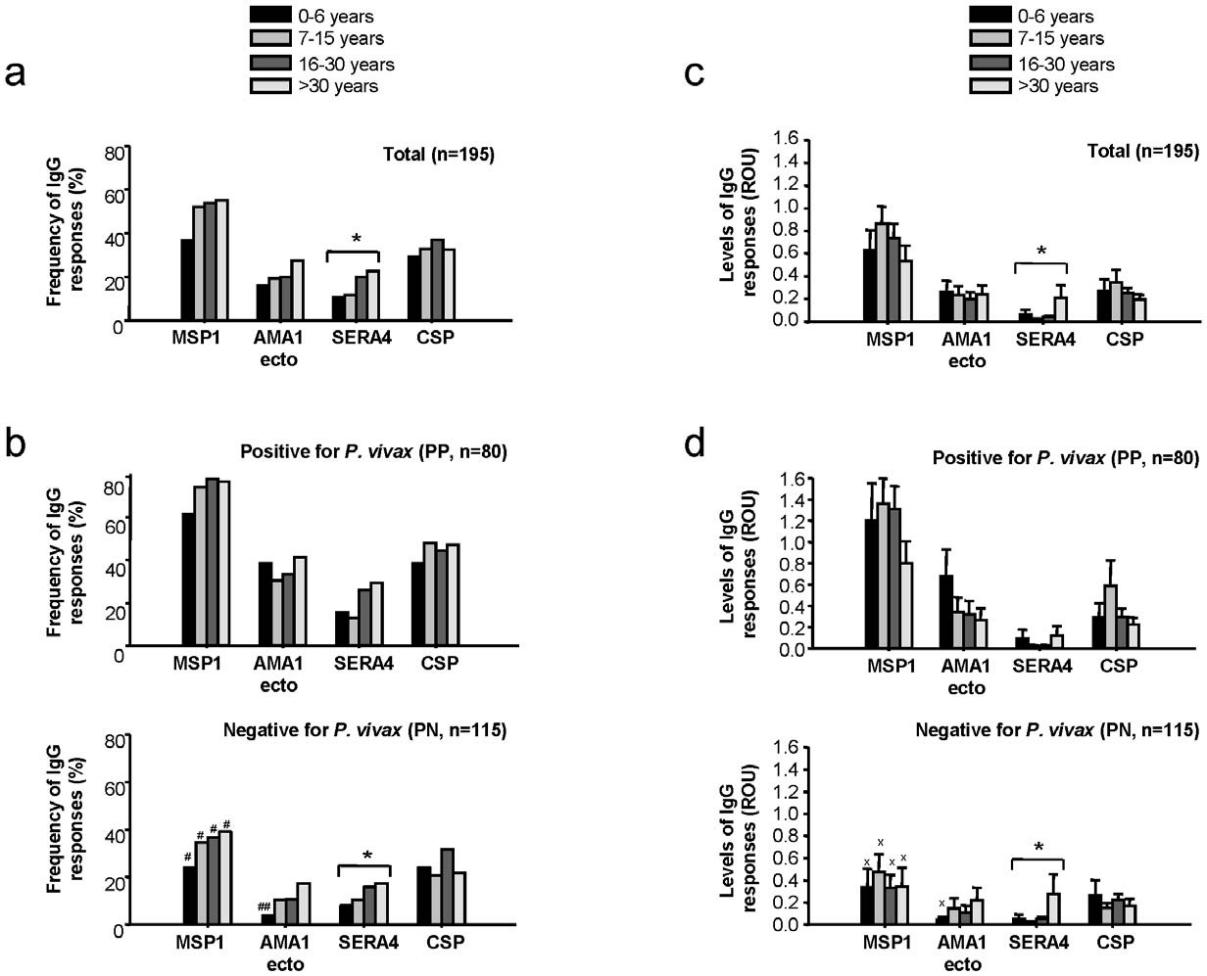
IgG antibody frequencies and levels according to age groups. IgG antibody frequencies in the total study population (*n* = 195) **(a)**, and in the blood smear positive (PP; *n* = 80) and blood smear negative (PN; *n* = 115) populations **(b)**. The number of individuals within each age group is shown in Table 1. # indicates significant differences between the PP and PN as assessed using the χ^2^ test (#*P*<0.05, and ##*P*<0.01). Total IgG antibody responses (ROU) to the four antigens grouped according to age for the total population (*n* = 195) **(c)**, blood smear positive (PP; n = 80) and negative (PN; n = 115) individuals **(d)**. x indicates significant differences in antibody levels (ROU) between blood smear positive and negative groups using multiple comparison analysis in conjunction with ANOVA (x, *P*<0.05). * indicates a significant correlation between increases in antibody frequencies, levels and age (r = 0.322, *P* = 0.0001 and r = 0.413, *P* = 0.001 in **(a, c)** and **(b, d)** respectively).

**Figure 3:**
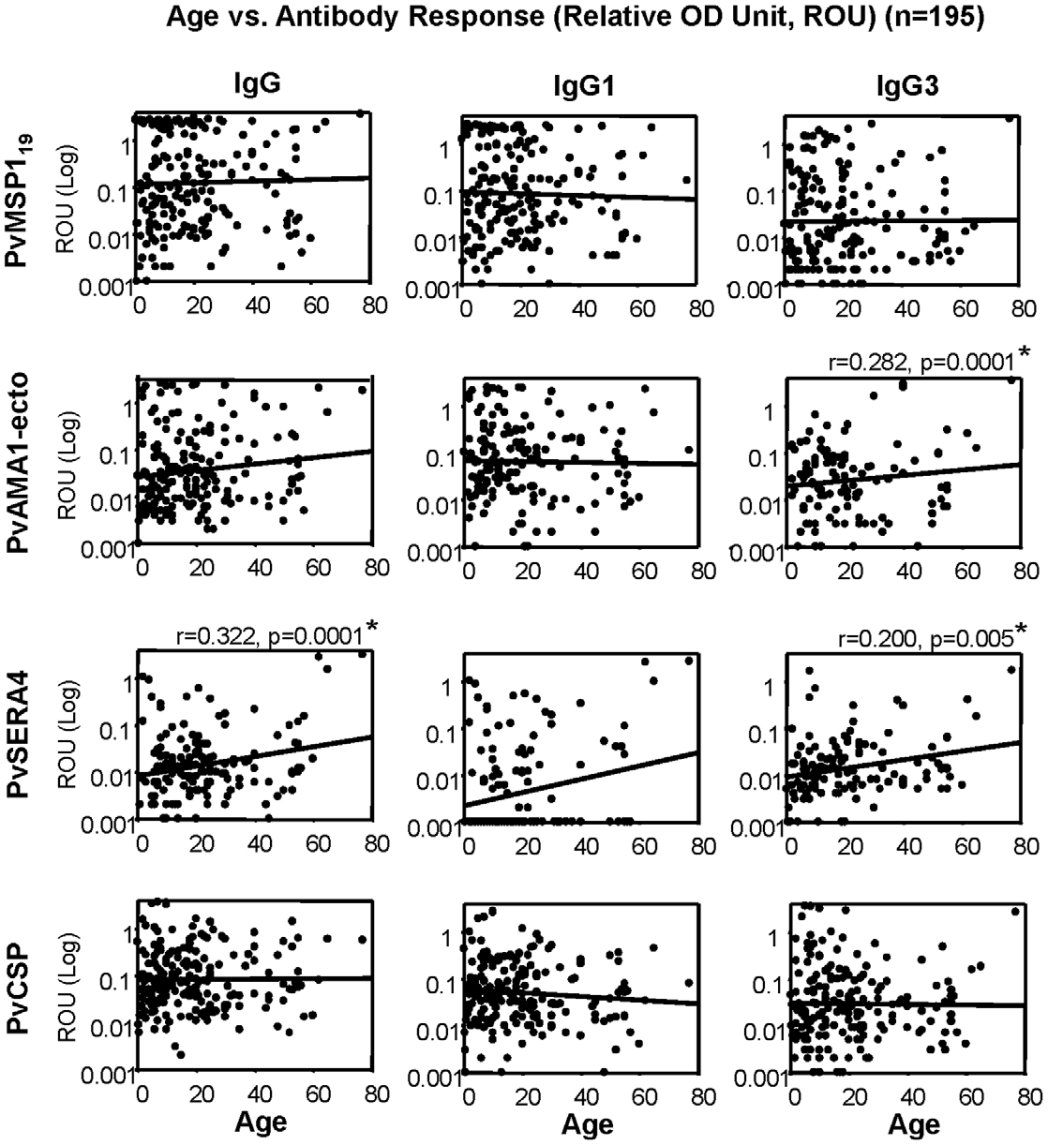
Correlation between age and total IgG, IgG1 and IgG3 antibody responses (ROU) to *P. vivax* MSP1_19_, PvAMA1-ecto, PvSERA4 and chimeric PvCSP antigens in all individuals (*n* = 195). Correlations were evaluated using Spearman's correlation test. Significantly correlated values were marked on the each figure (*).

We next examined the relationship between age and serum antibody responses, either between different age groups (Figure 2) or within the whole study population (Figure 3). With the exception of PvCSP, the frequency of IgG antibody responses to most of the antigens tended to increase with age (Figure 2a), while the serum levels did not differ significantly between the age groups (Figure 2c). Approximately 10–35% of individuals showed positive IgG antibody responses to at least one of the antigens by the age of 6, increasing to ∼25–60% after the age of 30 (Figure 2a). For the whole study population, the trend of increasing total IgG responses with age was most marked for PvSERA4 (r = 0.322; *P* = 0.0001) (Figure 3). IgG3 responses to PvSERA4 and PvAMA1-ecto also increased significantly with age (r = 0.200, *P* = 0.005 and r = 0.282, *P* = 0.0001, respectively; Figure 3).

In contrast, there was no correlation between IgG responses to any of the antigens and age in the PP population (*n* = 80; data not shown and Figure 2b). The increase in the IgG response was very rapid, even in the 0–6 age group, the exception being the IgG response to PvSERA4 (Figure 2b and Figure 2d). As expected, IgG responses (both in terms of frequency and serum levels) were lower in the PN population (*n* = 115; Figure 2b and Figure 2d; ^#^ *P*<0.05 and ^##^ *P*<0.01; χ^2^ test). However, despite the fact that IgG antibody responses were lower when compared with those in the whole study population or those in PP individuals (Figure 2d), 72% of PN individuals showed positive antibody responses (IgG and/or IgM) to at least one of the antigens (data not shown). Overall, IgG seropositivity against all the antigens studied (except PvCSP) in the PN population tended to increase with age (Figure 2b). The significant increase in the IgG response to PvSERA4 and the IgG3 response to PvAMA1-ecto with age (Figure 3) suggests that repeated exposure may be required to produce (and sustain) an increase in IgG responses to these two antigens. The IgG responses to most of the antigens (except PvSERA4) are apparent at a very early age; by contrast, IgG responses to PvSERA4 do not develop quickly after infection (s), but increases with age in concordance with PN status.

### Correlation between antibody levels and parasitemia

**Figure 4:**
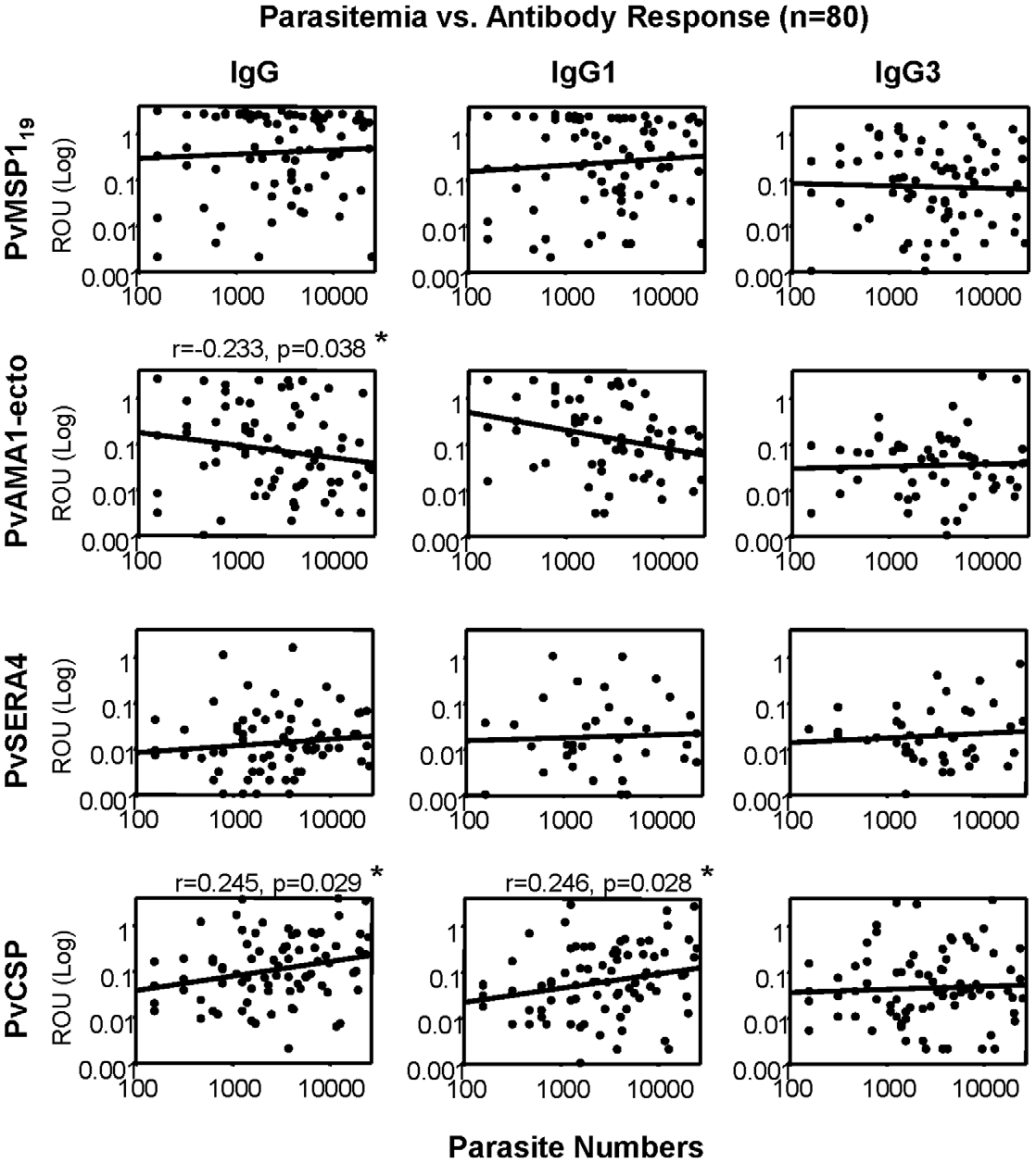
Correlation between parasite density and antibody levels. Total IgG, IgG1 and IgG3 antibody responses (ROU) to *P .vivax* MSP1_19_, PvAMA1-ecto, PvSERA4 and chimeric PvCSP antigens were measured in individuals with patent *P vivax* infection (*n* = 80). Correlations were evaluated using Spearman's correlation test. Significantly correlated values are marked on each figure (*).

We next evaluated the correlation between antibody responses and parasitemia during patent infection. Total IgG and IgG1 responses to PvCSP were positively correlated with parasitemia (r = 0.245, *P* = 0.029 and r = 0.246, *P* = 0.028, respectively; Figure 4). Conversely, IgG responses to PvAMA1-ecto were negatively correlated with parasitemia (r = −0.233, *P* = 0.038, Figure 4). There was no correlation between parasitemia and IgG3 levels for any antigen. These data support that PvCSP responses correlate well with parasite levels during acute infection [20]; however, the negative correlation between parasitemia and PvAMA1-ecto IgG responses may suggest an important role for high antibody levels to AMA1 in controlling parasitemia during *P. vivax* infection.

## Discussion

Understanding immunity to malaria parasites is crucial for successful interventions. Despite the introduction of extensive malaria elimination programs since the 1920s [18], *P. vivax* malaria has re-emerged in southeastern Turkey. To date, no study has been performed to understand the epidemiology and transmission dynamics of the *P. vivax* parasites in this region. Southeastern Turkey is unusual in that individuals are infected with *P. vivax* alone, with no co-infection by other malarial species. Thus, analysis of this region has clear implications for *P. vivax* biology and developing various control strategies such as vaccines. Serological parameters were shown in *P. falciparum* infections to offer an advantage for measuring endemicity and malaria transmission dynamics, because of overcoming sampling variations and the detection of persistent antibodies over months and years after infection [21]. We used a similar approach to get an overview/ rapid assessment of *P. vivax* transmission intensity. Likewise, this study is the first to analyze a range of serological parameters for individuals living in Sanliurfa, southeastern Turkey. Evaluations based on parasite prevalence, parasite exposure history and age may not be directly correlated with malaria protection but raise important clues for assessment in this unique malaria setting for *P. vivax* eventual monitoring and control.

The results of this study showed that IgG antibody responses to both pre-erythrocytic and erythrocytic antigens were closely related to a previous history of malaria infection. While only 10% of the actively infected population had a previous history of malaria infection, this increased three times (30.5%) in the PN population. This may indicate that one or two infections may induce naturally acquired immunity. Supporting this notion, IgG responses against *P. vivax* were positively correlated with age. Importantly, antibody levels to PvSERA4 increased with age which might have implications for future vaccine design. In addition, the PvAMA1 ectodomain portion may be a potential candidate vaccine antigen for reducing parasite levels. Whether these observations can robustly correlate with malaria protection, a future study should be able to address these using defined cohorts.

In agreement with previously published studies of *P. vivax* and *P. falciparum* infection, IgG responses to PvMSP1_19_ increased rapidly during the early ages of infection and were sustained over a long period of time [21], [22], [23], [24], although there was no significant correlation between age, parasite levels, and antibody responses to PvMSP1_19_. However, in an earlier pilot study, we observed higher sero-reactivity (53.6% IgG responses to PvMSP1_19_) to the C-terminal region of the PvMSP1 antigen (produced in *Saccharomyces cerevisiae*) in the PP population, which was negatively correlated with parasite levels [16]. In the current study, we did not find any correlation between antibody responses to PvMSP1_19_ and parasite number, suggesting the possibility of allelic polymorphisms in the antigen/parasites. In fact, in a recent study, we did find eight substitutions in the PvMSP1 gene that were unique to the Turkish *P. vivax* population and one of them (D/E at 1706 in the C-terminal 19-kDa region) was previously unidentified [17]. To understand whether this unique D/E substitution has impact on the antibody responses and parasite levels, we measured total IgG levels in a limited numbers of infected serum bearing the D/E substitution. Preliminary results suggested that there was an inverse correlation between anti-PvMSP1_19_ IgG levels and parasite numbers in individuals infected with E haplotype substitutions, but not D haplotype substitutions (data not shown, *unpublished observation*). It is also noteworthy that other studies from Brazil and Papua New Guinea reported that different individuals show naturally acquired IgG responses to different regions of PvMSP1 [25], [26]. Further confirmatory tests would be needed to substantiate further the effect of different haplotypes/allelic polymorphisms to antibody responses.

Similar to anti-PvMSP1_19_ responses, antibody levels to chimeric PvCSP antigen immediately increased in younger individuals, but the response was weaker than that for PvMSP1_19_, even during patent infection (Figure 2c). However, these responses did not completely disappear, and were even sustained in PN individuals with no evidence of prior malaria infection (presumably naïve; Figure 1c). One possible explanation for this might be an ongoing undetectable level of transmission by mosquitoes in this population as reported recently, suggesting the occurrence of *P. vivax* infection with ultra low-level parasitemia, which may maintain transmission levels, even under controlled-radical therapy [27]. Although we were technically unable to confirm the microscopically *P. vivax*-negative individuals by PCR in the present dataset, based on our experience from this region, genus-specific Real-Time PCR could only detect 4.5% parasite positivity over microscopy even in the malaria-suspected symptomatic patients (Yuksel and Zeyrek, *unpublished observations*). Therefore, it seems unlikely that more than 4.5% of malaria history negative PN group (presumably naïve) individuals have undetectable level of parasitemia. We cannot exclude, however, another possibility of *P. vivax* infections causing relapses due to the presence of hypnozoites, even in the absence of mosquito bites, because of ineffective drug treatment [28], [29]. To date, it has always been a technical difficulty to address how hypnozoites/ relapses could influence serological profiles [30]. Currently there is no molecular tool to discriminate re-infection versus relapse during *P. vivax* infection and this certainly limits meaningful evaluations to specific populations as re-infection probabilities may exceed 20% per year [31]. All together, these possibilities may explain the higher IgG seropositivity in parasite and presumably malaria history negative individuals.

We observed antibody responses to PvAMA1-ecto antigens in this region, although low in frequency and accompanied by low serum antibody levels. Children infected at a younger age (<6 years old) developed rapid antibody responses to AMA1-ecto antigen, but these responses were not seen after 6 years of age. However, the current study provides important evidence that IgG antibody responses to PvAMA1-ecto domain are significantly increased in non-infected individuals with a prior history of malaria infection. In addition, the IgG levels to PvAMA1-ecto were negatively correlated with parasite levels, which collectively may suggest that antibodies to PvAMA1-ecto are important and maybe closely related to protection [32]. These observations certainly require additional defined-cohort studies to address antibody levels-protection correlations.

This is the first report analyzing anti-*P. vivax* SERA4 responses. The serine repeat antigen (SERA) is an abundant asexual blood-stage antigen primarily expressed by *Plasmodium* parasites during late trophozoite and schizont stages [33], [34], [35]. In the case of *P. falciparum*, upon schizont rupture theabundant Pf SERA gene family member, SERA5, is processed to yield a 47 kDa N-terminal, a 50 kDa central, an 18 kDa C terminal, and a 6 kDa domain [34], [35]. The N-terminal 47-kDa domain of *P. falciparum* serine repeat antigen 5 (PfSERA5) has already been exploited as a potential candidate vaccine [34]. An epidemiological study in a malaria hyper-endemic area of Uganda revealed that naturally-induced IgG responses to the N-terminal 47 kDa domain were positively correlated with increased levels of protective immunity in adults [36]. In earlier studies with *P. vivax*, the highest transcription of PvSERA4 in all field isolates [33] parallels that of PfSERA5. This was our basis on the selection of the expressed region of PvSERA4, which was similar in amino acid sequence to the 47-kDa domain of PfSERA5. In this study, we observed IgM responses to PvSERA4 in 50% of individuals, while only 16% showed IgG responses. Given that higher IgG levels were more frequent in the PN population only after re-exposure to malaria and after certain ages, there is a possibility that seroconversion from IgM antibodies to IgG for anti-PvSERA4 antibodies, are somehow not occurring properly. This is an interesting hypothesis that needs further investigation. Similar to the limited PvMSP1_19_ gene polymorphisms observed in this region [17], initial analysis of the PvSERA4 gene in Sanliurfa isolates also revealed limited polymorphisms at the N-terminal region when compared with parasite isolates from southeast Asia (Arisue N and Horii T, *unpublished observations*). However, the polymorphic nature of the recombinant PvSERA4 antigen used in the present study (and the other antigens to a similar extent) may also be one reason that we detected low IgG antibody responses in this region. This, too, requires further investigation.

This study constitutes a first seroepidemiological analysis/survey of antibodies to a variety of blood and pre-erythrocytic stage malarial antigens and provides valuable information in their relation to the age of the individuals living in a sole *P. vivax* endemic region. However, we are far from the conclusion that naturally acquired antibodies to these relatively small numbers of vivax proteins could establish malaria immunity and/or protection. Apparently, new tools such as protein microarrays which represent at least 20% of the parasite proteome are needed to evaluate naturally acquired antibodies which may be associated with naturally acquired vivax malaria immunity in this setting as similar to recently described study for falciparum malaria [37].

Nevertheless, our study offers valuable insights to those designing vaccines/drugs especially in the settings where elimination programs have been launched [38].

## Materials and Methods

### Ethics Statement

All samples were collected after written informed consent was obtained from the patients (or the parents of individuals under 18 years of age), prior to anti-malarial treatment when appropriate. All clinical investigations were conducted according to the principles expressed in the Declaration of Helsinki. Authorization was obtained from the Turkish Ministry of Health, Sanliurfa Bureau, and ethical approval was obtained from the Research Institute for Microbial Diseases, Osaka University.

### Study population and demographic characteristics

One hundred and ninety-five serum samples were collected from individuals in Sanliurfa province (the towns of Siverek and Harran) in southeastern Turkey during 2004 and 2008. The samples were collected at the peak of the malaria season (July to November) and kept at −20°C until use. All 69 serum samples collected in 2004 were found to be infected with patent *P. vivax* malaria (as diagnosed by microscopy) [16]. The unbiased, age and gender matched 126 serum samples from 2008 were collected during active surveillance (house-to-house screening) (Table S1). Of these serum samples, 11 (8.7%) were confirmed *P. vivax*-infected using thick blood smears after Giemsa staining. The level of parasitemia (asexual parasites/µL blood) was determined as previously described [15]. Statistical analysis showed no significant difference between the two patient groups in terms of population demographics and antibody responses (*n* = 69 from 2004 and *n* = 11 from 2008) (data not shown); and hence, the two sets of samples were grouped together as “microscopically positive for *P. vivax* parasites” (*n* = 80) (see Table S1).

The mean age of the sample population was 20.5±16.2 years [0–77 years] (mean ± SD [range]), and 47.7% of the subjects were male. The samples were divided into four age groups: 0–6 years old (19.5%), 7–15 years old (26.7%), 16–30 years old (33.3%), and >31 years old (20.5%). Table S1 shows the baseline characteristics of the study subjects. The study included individuals infected with *P. vivax* (*P. vivax*-positive (PP) according to blood smear results; *n* = 80) and individuals living in the malaria-endemic area but not infected with *P. vivax* at the time of sampling (*P. vivax* negative (PN) according to blood smear results; *n* = 115). The mean (mean ± SD [range]) age of the two groups was 21.1±16 [0–65] years and 20.4±16.5 [0–77] years, respectively, and 56.2% and 41.7% of the respective groups were male. There was a significant difference in body temperature between the PP and PN groups (37.9±0.522°C *vs.* 36.7±0.513°C, respectively; *P*<0.0001). The mean parasite density was 5502±6386 [160–25560] parasites/µL. The majority of the patients (46.3%) had parasite densities between 1001 and 5000 parasites/µL, whereas only 32.5% of the patients had heavy parasitemia (> 5000 parasites/µL) and 21.3% had very low parasitemia (< 1000 parasites/µL). Mean hemoglobin, hematocrit concentrations, and mean white blood cell counts did not vary significantly between the PP and PN groups (Table S1).

### Determination of malaria infection history

Disclosure of malaria infection is compulsory in Turkey and intervention is controlled by the Turkish Ministry of Health, alongside local national malaria control centers, in accordance with the World Health Organization criteria for malaria diagnosis and treatment. Questionnaires filled out by the subjects or their parents ascertained whether they were previously diagnosed and treated for malaria at any of the local malaria control centers. The answers were cross-checked against the records from the malaria control centers. The results showed that those individuals with a history of previous malaria infection had only a single exposure (> 95%).

### Recombinant *P. vivax* antigens

PvMSP1_19_, PvAMA1-ecto, and PvCSP were expressed using a wheat germ cell-free protein translation system (CellFree Sciences, Matsuyama, Japan) [39], whereas PvSERA4 was cloned and expressed in *E. coli* as a histidine tagged fusion protein. The PvCSP was designed as recombinant chimeric protein that presumably cover vivax parasite population globally as previously described in detail [40]. PvAMA1-ecto encompasses ectodomain of PvAMA1 (Glu_77_ to Gln_484_ based on the *Sal*I sequence, PVX_ 092275). Both gene fragments were cloned into pEU-E01-His-TEV-N2 plasmid for the wheat germ cell-free system (CellFree Sciences).

PvSERA4 gene sequence corresponding to amino acid regions Val_19_ to Lys_352_ was amplified from gDNA of *Sal*I after comparing sequences with NICA and Chesson. The N-terminal position and prediction of peptide cleavage site was determined using SignalP 3.0 [41]. The C-terminal position was determined by alignment with PfSERA5 N-terminal domain. After alignment and intron identification/localization, introns were spliced by overlapping PCR. The amplified fragment was ligated to pET-15b, and the resulting plasmid with hexa-His-tag was transformed to *E. coli* Rosetta-gami B (DE3) pLysS (Novagen, San Diego, CA). Protein expression was induced by the addition of isopropyl-β-D-thiogalactopyranoside to a final concentration of 0.5 mM. The recombinant protein was extracted into soluble fraction with BugBuster Master Mix (Novagen) and purified using a His GraviTrap (GE Healthcare). All antigens were stored at −80°C until assayed.

### Measurement of serum antibody levels by ELISA

The levels of human IgG, IgM, IgG1, IgG2, IgG3, and IgG4 antibodies against PvMSP1_19_, PvCSP, PvAMA1-ecto and PvSERA4 were measured by ELISA as previously described [42], [43]. Briefly, 96-well microtiter plates (Maxisorb, Nunc, Denmark) were coated with either 0.5 or 1 µg/ml of each of the recombinant antigens in bicarbonate buffer and incubated overnight at 4°C. After blocking, serum samples (100 µl, diluted 1∶100 in a PBS-Tween buffer containing 5% skim milk) were added to the wells. The serum dilution and the amount of coated antigen were confirmed in pilot experiments as optimal for the accurate measurement of antibody levels. After washing four times, the plates were incubated with horseradish peroxidase-conjugated secondary antibodies to human IgG, IgM and the four IgG subclasses (1∶1000; Zymed, Carlsbad, USA) at room temperature for 2 h. The plates were washed four times and 3, 3′, 5-5′-tetramethylbenzidine (Sigma) was added to each well and incubated in the dark at room temperature. The optical density was then measured at 450 nm. Each sample was assayed in duplicate and each plate contained “blank” wells and control sera. The OD values were normalized according to cut-off value and expressed as relative OD units (ROU). Cut-off values were set at three standard deviations above the mean OD_450_ measured using sera from 20 Japanese blood donors with no history of malaria exposure. After subtracting the cut-off values, any ROU measurements higher than 0.01 were considered positive. The cut-off values were as follows: anti-PvMSP1_19_-IgG, IgM, IgG1, IgG2, IgG3 and IgG4: 0.094, 0.345, 0.052, 0.324, 0.022 and 0.367, respectively; anti-PvAMA1-ecto-IgG, IgM, IgG1, IgG2, IgG3 and IgG4: 0.145, 0.542, 0.204, 0.32, 0.064 and 0.059, respectively; anti-PvSERA4-IgG, IgM, IgG1, IgG2, IgG3 and IgG4: 0.03, 0.405, 0.019, 0.062, 0.0418 and 0.01, respectively; and anti-PvCSP-IgG, IgM, IgG1, IgG2, IgG3 and IgG4: 0.141, 0.775, 0.14, 0.41, 0.05 and 0.1, respectively.

### Statistical analysis

Data from 2004 and 2008 were pooled and/or divided into two groups: blood smear*-*positive and blood smear-negative for *P. vivax*. The antibody levels in all age groups, and in the blood smear-positive and -negative groups, were compared using the X^2^ test. Differences in antibody levels (expressed as ROU) between the groups were analyzed using multiple comparison tests in conjunction with ANOVA. Spearman's rank correlation was used to evaluate correlations between the variables. A *P* value < 0.05 was considered significant. All statistical analysis was performed using the SPSS statistical package (version 10.0; SPSS, Chicago, IL, USA).

## Supporting Information

Table S1 **Demographic characteristics of the study population.** * Significant values were determined between microscopically confirmed parasite positive vs. parasite negative populations by Student's t-test and X^2^- tests with a level of significance set at P<0.05. ^a^ Statistically significant by Student's t-test.
(DOC)

## References

[1] Guerra CA, Snow RW, Hay SI (2006). Mapping the global extent of malaria in 2005.. Trends Parasitol.

[2] Mueller I, Galinski MR, Baird JK, Carlton JM, Kochar DK (2009). Key gaps in the knowledge of Plasmodium vivax, a neglected human malaria parasite.. Lancet Infect Dis.

[3] Galinski MR, Barnwell JW (2008). Plasmodium vivax: who cares?. Malar J.

[4] Brown GV, Moorthy VS, Reed Z, Mendis K, Arevalo-Herrera M (2009). Priorities in research and development of vaccines against Plasmodium vivax malaria.. Vaccine.

[5] Mendis K, Sina BJ, Marchesini P, Carter R (2001). The neglected burden of Plasmodium vivax malaria.. Am J Trop Med Hyg.

[6] Michon P, Cole-Tobian JL, Dabod E, Schoepflin S, Igu J (2007). The risk of malarial infections and disease in Papua New Guinean children.. Am J Trop Med Hyg.

[7] Lin E, Kiniboro B, Gray L, Dobbie S, Robinson L (2010). Differential patterns of infection and disease with P. falciparum and P. vivax in young Papua New Guinean children.. PLoS One.

[8] Luxemburger C, Nosten F, White NJ (1999). Naturally acquired immunity to vivax malaria.. Lancet.

[9] Mueller I, Moorthy VS, Brown GV, Smith PG, Alonso P (2009). Guidance on the evaluation of Plasmodium vivax vaccines in populations exposed to natural infection.. Vaccine.

[10] Kochar DK, Tanwar GS, Khatri PC, Kochar SK, Sengar GS (2010). Clinical features of children hospitalized with malaria--a study from Bikaner, northwest India.. Am J Trop Med Hyg.

[11] Chuangchaiya S, Jangpatarapongsa K, Chootong P, Sirichaisinthop J, Sattabongkot J (2010). Immune response to Plasmodium vivax has a potential to reduce malaria severity.. Clin Exp Immunol.

[12] Douglas NM, Nosten F, Ashley EA, Phaiphun L, van Vugt M (2011). Plasmodium vivax Recurrence Following Falciparum and Mixed Species Malaria: Risk Factors and Effect of Antimalarial Kinetics.. Clin Infect Dis.

[13] Snounou G, White NJ (2004). The co-existence of Plasmodium: sidelights from falciparum and vivax malaria in Thailand.. Trends Parasitol.

[14] Lin JT, Bethell D, Tyner SD, Lon C, Shah NK (2011). Plasmodium falciparum gametocyte carriage is associated with subsequent Plasmodium vivax relapse after treatment.. PLoS One.

[15] Zeyrek FY, Kurcer MA, Zeyrek D, Simsek Z (2006). Parasite density and serum cytokine levels in Plasmodium vivax malaria in Turkey.. Parasite Immunol.

[16] Zeyrek FY, Babaoglu A, Demirel S, Erdogan DD, Ak M (2008). Analysis of naturally acquired antibody responses to the 19-kd C-terminal region of merozoite surface protein-1 of Plasmodium vivax from individuals in Sanliurfa, Turkey.. Am J Trop Med Hyg.

[17] Zeyrek FY, Tachibana S, Yuksel F, Doni N, Palacpac N (2010). Limited polymorphism of the Plasmodium vivax merozoite surface protein 1 gene in isolates from Turkey.. Am J Trop Med Hyg.

[18] Kratz FW, Bridges CB (1956). Malaria control in Turkey.. Public Health Rep.

[19] Corran P, Coleman P, Riley E, Drakeley C (2007). Serology: a robust indicator of malaria transmission intensity?. Trends Parasitol.

[20] John CC, Moormann AM, Pregibon DC, Sumba PO, McHugh MM (2005). Correlation of high levels of antibodies to multiple pre-erythrocytic Plasmodium falciparum antigens and protection from infection.. Am J Trop Med Hyg.

[21] Drakeley CJ, Corran PH, Coleman PG, Tongren JE, McDonald SL (2005). Estimating medium- and long-term trends in malaria transmission by using serological markers of malaria exposure.. Proc Natl Acad Sci U S A.

[22] Lim KJ, Park JW, Yeom JS, Lee YH, Yoo SB (2004). Humoral responses against the C-terminal region of merozoite surface protein 1 can be remembered for more than 30 years in persons exposed to Plasmodium vivax.. Parasitol Res.

[23] Wickramarachchi T, Illeperuma RJ, Perera L, Bandara S, Holm I (2007). Comparison of naturally acquired antibody responses against the C-terminal processing products of Plasmodium vivax Merozoite Surface Protein-1 under low transmission and unstable malaria conditions in Sri Lanka.. Int J Parasitol.

[24] Rodrigues MH, Cunha MG, Machado RL, Ferreira OC, Rodrigues MM (2003). Serological detection of Plasmodium vivax malaria using recombinant proteins corresponding to the 19-kDa C-terminal region of the merozoite surface protein-1.. Malar J.

[25] Soares IS, Levitus G, Souza JM, Del Portillo HA, Rodrigues MM (1997). Acquired immune responses to the N- and C-terminal regions of Plasmodium vivax merozoite surface protein 1 in individuals exposed to malaria.. Infect Immun.

[26] Fernandez-Becerra C, Sanz S, Brucet M, Stanisic DI, Alves FP (2010). Naturally-acquired humoral immune responses against the N- and C-termini of the Plasmodium vivax MSP1 protein in endemic regions of Brazil and Papua New Guinea using a multiplex assay.. Malar J.

[27] Van den Eede P, Soto-Calle VE, Delgado C, Gamboa D, Grande T (2011). Plasmodium vivax sub-patent infections after radical treatment are common in Peruvian patients: results of a 1-year prospective cohort study.. PLoS One.

[28] Imwong M, Snounou G, Pukrittayakamee S, Tanomsing N, Kim JR (2007). Relapses of Plasmodium vivax infection usually result from activation of heterologous hypnozoites.. J Infect Dis.

[29] Wells TN, Burrows JN, Baird JK (2010). Targeting the hypnozoite reservoir of Plasmodium vivax: the hidden obstacle to malaria elimination.. Trends Parasitol.

[30] Kirchgatter K, del Portillo HA (1998). Molecular analysis of Plasmodium vivax relapses using the MSP1 molecule as a genetic marker.. J Infect Dis.

[31] Baird JK (2009). Resistance to therapies for infection by Plasmodium vivax.. Clin Microbiol Rev.

[32] Mufalo BC, Gentil F, Bargieri DY, Costa FT, Rodrigues MM (2008). Plasmodium vivax apical membrane antigen-1: comparative recognition of different domains by antibodies induced during natural human infection.. Microbes Infect.

[33] Palacpac NM, Leung BW, Arisue N, Tanabe K, Sattabongkot J (2006). Plasmodium vivax serine repeat antigen (SERA) multigene family exhibits similar expression patterns in independent infections.. Mol Biochem Parasitol.

[34] Horii T, Shirai H, Jie L, Ishii KJ, Palacpac NQ (2010). Evidences of protection against blood-stage infection of Plasmodium falciparum by the novel protein vaccine SE36.. Parasitol Int.

[35] Palacpac NM, Arisue N, Tougan T, Ishii KJ, Horii T (2011). Plasmodium falciparum serine repeat antigen 5 (SE36) as a malaria vaccine candidate.. Vaccine.

[36] Okech B, Mujuzi G, Ogwal A, Shirai H, Horii T (2006). High titers of IgG antibodies against Plasmodium falciparum serine repeat antigen 5 (SERA5) are associated with protection against severe malaria in Ugandan children.. Am J Trop Med Hyg.

[37] Crompton PD, Kayala MA, Traore B, Kayentao K, Ongoiba A (2010). A prospective analysis of the Ab response to Plasmodium falciparum before and after a malaria season by protein microarray.. Proc Natl Acad Sci U S A.

[38] Cook J, Reid H, Iavro J, Kuwahata M, Taleo G (2010). Using serological measures to monitor changes in malaria transmission in Vanuatu.. Malar J.

[39] Tsuboi T, Takeo S, Iriko H, Jin L, Tsuchimochi M (2008). Wheat germ cell-free system-based production of malaria proteins for discovery of novel vaccine candidates.. Infect Immun.

[40] Rui E, Fernandez-Becerra C, Takeo S, Sanz S, Lacerda MV (2011). Plasmodium vivax: comparison of immunogenicity among proteins expressed in the cell-free systems of Escherichia coli and wheat germ by suspension array assays.. Malar J.

[41] Emanuelsson O, Brunak S, von Heijne G, Nielsen H (2007). Locating proteins in the cell using TargetP, SignalP and related tools.. Nat Protoc.

[42] Coban C, Philipp MT, Purcell JE, Keister DB, Okulate M (2004). Induction of Plasmodium falciparum transmission-blocking antibodies in nonhuman primates by a combination of DNA and protein immunizations.. Infect Immun.

[43] Culleton R, Ndounga M, Zeyrek FY, Coban C, Casimiro PN (2009). Evidence for the transmission of Plasmodium vivax in the Republic of the Congo, West Central Africa.. J Infect Dis.

