# Supplementary material for: Serologic Markers in Relation to Parasite Exposure History Help to Estimate Transmission Dynamics of Plasmodium vivax
Source: PLoS One. 2011 Nov 29;6(11):e28126. doi: 10.1371/journal.pone.0028126 (PMC3226671; doi:10.1371/journal.pone.0028126)
Supplement: Table S1 — Demographic characteristics of the study population. * Significant values were determined between microscopically confirmed parasite positive vs. parasite negative populations by Student's t-test and X2- tests with a level of significance set at P<0.05. a Statistically significant by Student's t-test. (DOC) [file pone.0028126.s001.doc]

**Supplementary Table** 1.

|  | **Blood smear positive**  **(PP) (n=80)** | **Blood Smear**  **negative**  **(PN) (n=115)** | **Test and**  **P-values^*^** | **Total**  **(n=195)** |
| --- | --- | --- | --- | --- |
| **Age (Mean ± SD) (min-max)**    **0-6 years**  **7-15 years**  **16-30 years**  **>30** | 21.1±15.8 (0-65)  13 (16.3%)  23 (28.8%)  27 (33.8%)  17 (21.3%) | 20.0±16.5 (0-77)  25 (21.7%)  29 (25.2%)  38 (33%)  23 (20%) | X^2^= 0.99 P=0.80 | 20.5±16.2 (0-77)  38 (19.5%)  52 (26.7%)  65 (33.3%)  40 (20.5%) |
| **Sex**  **Male**  **Female** | 45 (56.2%)  35 (43.8%) | 48 (41.7%)  67 (58.3%) | X^2^= 3.98, P=0.05 | 93 (47.7%)  102 (52.3%) |
| **Temperature at admission (^0^C) (Mean ± SD)** | 37.9 ± 0.522 | 36.7 ± 0.513 | t= 7.31 P<0.0001^a^ | 36.8 ± 0.61 |
| **Wbc (x10^3^/mm^3^) (Mean ± SD)** | 7.5 ± 4.8 | 6.9 ± 3.5 | t= -0.42 P=0.67 | 6.9 ± 3.6 |
| **Hematocrit (%) (Mean ± SD)** | 44.2 ± 8.3 | 39.2 ± 7.5 | t= -1.70 P=0.09 | 39.5 ± 7.6 |
| **Hemoglobin (g/dL) (Mean ± SD)** | 13.3 ± 2.3 | 13.4 ± 1.6 | t= 0.05 P=0.95 | 13.4 ± 1.7 |
| **Parasite density**  **(Mean ± SD )**  **(min-max)**  **150-1000**  **1001-5 000**  **>5 001** | 5502 ± 6386  (160-25560)  17 (21.3%)  37 (46.3%)  26 (32.5%) | 0 | N.A. | 5502 ± 6386  (160-25560) |
